# Peri-Nephritic Abscess

**Published:** 1888-04

**Authors:** W. H. Heath

**Affiliations:** Surgeon to the Sisters of Charity Hospital, Buffalo


THE BUFFALO MEDICAL AND SURGICAL JOURNAL

VOL. XXVII. APRIL, 1888. No. 9

### Original Communications.

### *PERI-NEPHRITIC ABSCESS.*

BY W. H. HEATH, M. D.,
Surgeon to the Sisters of Charity Hospital, Buffalo.

The cellulo-adipose tissue in which the kidney is imbedded is occasionally the seat of inflammation and suppuration. It is rather an uncommon affection, often difficult of recognition and generally dangerous. Depending on their mode of origin, these abscesses are arranged in three groups, viz.:

*Primary.*—Those arising independent of disease of the kidney or other organ. These sometimes follow exposure, but are most frequently caused by traumatism, strains, particularly while in a stooping position, and especially in persons in whom there appears a predisposition to suppuration, as evinced by slow healing of wounds, eruption of boils, whitlow, etc.

*Secondary.*—Those consecutive to disease of the kidney. In general, all suppurative inflammations of kidney or pelvis, if continued, are very likely to set up inflammation and abscess by extension, while extravasations of urine or pus are certain to.

*The third class* are those due to extension from other parts, as from carious vertebræ and, in females, from pelvic cellulitis.

Chronic inflammations and operations upon the lower urinary organs are particularly liable to be followed by this abscess, and the possibility of its existence should be considered in all obscure cases or complications of disease or injury in this region.

Clinically, they may be arranged in three groups. The *acute*, having a rapid onset course and pus-formation, and which are ushered in with the usual constitutional evidence of severe inflammatory injury and, locally, by pain, swelling in the affected loin; *the sub-acute, or chronic*, characterized by a slow, indolent, subtle course; and, thirdly, those cases where the symptoms are masked by the disease they are consecutive to, and, therefore, will not be considered.

The general symptoms vary in degree as in other phlegmons. In acute cases, rigors or chill, high temperature, derangement of secretions, etc., accompany local symptoms of proportionate severity, and the diagnosis is not different. In *sub-acute* or chronic cases, however, until suppuration occurs, and even subsequently, the constitutional evidence may be simply a fever of so varying a grade and character as to lead to the suspicion of enteric or malarial difficulty, or a condition may exist merely of a general wasting and weakness—progressive loss of health—the local evidence of serious organic trouble in the loin being so slight or vague as to attract little or no attention.

The local symptoms are those usual to deep suppurations, modified, however, by anatomical peculiarities and the severity of the cases.

Pain, the most important of all symptoms, is almost always present in some degree—in acute cases, is intense; in sub-acute, may be slight, or disappear and return—and is characterized by being reflected over the ilio-lumbar nerves, to the groin, scrotum, penis, leg, knee or hip, etc. It is, of course, aggravated by movement, and peculiarly by defecation, in consequence of the proximity of the colon to the inflamed area and the particular muscular action called for.

Swelling is preceded by tenderness and sense of tension, and consists more in an obliteration of the flank than the formation of a distinct tumor. Redness is only present late, when the overlying tissues are involved, and fluctuation is difficult to obtain. Lameness, from contraction of the psoas, and lateral inclination of the body are frequently early and important symptoms. This flexion of the thigh is rather characteristic, and, when early, is due to reflex irritation, later, to infiltration and inflammation of muscle. Constipation is almost always present, for the reasons stated.

In consequence of the reciprocal action between the upper and lower urinary organs, frequent micturition occurs and may exist from the beginning. The pus often has a fecal stench, from proximity to the bowels, and varies from an ounce to many pints. When it contains urine, is evidence of the secondary nature of the malady. The urine itself furnishes important evidence of anteceding renal trouble.

The dangers and complications of the disease arise from wandering of pus, in seeking its own way out, and which it does in accordance with strict anatomical rules. The kidney, which normally floats to a certain extent, becomes fixed—set as it were—from inflammation, effusion and pressure of the pus. Depending, therefore, upon the relation of the pus sac and point of fixation, it may burst into any of the adjacent organs, or, by following the ramifications of the cellular tissue back of peritoneum, make its way to the pelvis, and finally appear in Scarpa's triangle. Cases are recorded of pus finding an exit by opening into the colon, vagina, bladder, stomach, lungs, pleura and into pelvis of kidney itself. Penetration into lung is the most frequent complication, and certainly the most insidious and dangerous, except into the peritoneum, which, however, as these abscesses are generally posterior to the kidney and also the colon, is rare. When opening external—which is the most favorable course—it follows the connective tissue between the latissimus dorsi and external oblique, beyond the quadratus, in the position occupied by lumbar hernia.

Such complications are not invariably fatal, and are characterized by symptoms which invite attention to the region invaded, and by coincident diminution of local pain and swelling. Thus, when bursting into the colon, pus is found in the stools; into the stomach, it is vomited; when into the lung, dyspnœa, suffocation and copious purulent expectoration ensue; if into the pleural cavity, physical signs of empyema develop.

The following cases illustrate the salient features:

1. J. B.; saltwater seaman; twenty-six years. No particular family history. Had syphilis and gonorrhœa. Admitted to hospital with a fistulous opening in left loin, discharging three or four ounces of pus daily, and which he stated was "where the doctor in the West Indies had opened a large gathering a short time before his return to America." He stated, further, that he had "had a hard time with his back" previous to the gathering, the character of which could not be exactly determined from his remarks.

He gave no history of renal calculi or any inflammatory disease of the kidney; his urine was normal, and none escaped through the sinus, nor could he specify any particular injury to his back, but considered his trouble the result of hard work on long voyages. His other symptoms were lameness and stiffness in left leg, inability to lay on same side or sit upright, poor appetite, diarrhœa, alternating with constipation, emaciation, etc.

He was placed upon good food and tonics, the sinus enlarged, scraped, the abscess cavity drained and washed daily. Exploration failed to detect anything abnormal with the kidney, necrosed vertebræ, or foreign body. After a slight improvement in his general condition, he became worse. Fever developed, followed by pain, swelling and tenderness in right loin, which was opened, drained and washed. It was soon discovered that both abscesses communicated, and his condition steadily ran down. Shortly before he passed out of my hands, gastric and intestinal derangement became more marked, with bronzing, first, of skin over loins, and gradually of face, mucous membrane of mouth and other parts, showing implication of the supra-renal bodies. Neither fistulæ had healed, and his death was a question of but a few weeks.

2. Michael Boyle; thirty-six; seaman. Family history not considered. Habits bad. Admitted for gonorrhœa and cystitis, with a urethra already the seat of old and bad stricture in membranous portion. He complained also of backache, which he attributed to a fall received in a drunken row some time—eight weeks—previous. Urine was ammoniacal, contained mucus and pus in considerable quantity. He was a little feverish, looked sick, and walked stiff and lame, in consequence, as he said, of rheumatism. His treatment was directed solely to subduing the urethritis and cystitis, the possibility of lumbar abscess not being thought of at the time, that class of hard-laboring and dissipated men so often complaining of their backs. After some improvement in his local disease, his stricture was attacked by gradual dilatation, and the bladder by irrigation, with benefit, without, however, freeing or even diminishing the pus in urine, and he appeared sicker than he ought to. This, together with his keeping to bed during the day, increase of pain in back, inability to lay on his side, and the fact of his having had some chills, which up to then were considered urethral, directed my attention to his loins, the right one of which was found to be slightly full and tender. He also had complained of constipation and pain in defecating, but it was supposed to be due to prostratitis, and not much further thought given to it. The loin was aspirated, and a bottle of pus, free from urinary odor, withdrawn. The case was then considered one of pyelitis with peri-nephritic abscess, and the treatment changed. A free incision and exploration was made, followed by drainage and washing. After some three months, the cavity finally closed entirely, and the man one day left without permission, but with some pyelitis left, as shown by a small quantity of pus voided with his urine.

3. Mrs. B.; twenty-five; married, no children; school-teacher. History good and herself well, up to about three years ago, when she suffered more or less from vague abdominal cramps, not characteristic of anything, but likely renal in nature. During this time, she describes her urine as being generally colored and gritty; red deposits. Frequent micturition developed and, later, irregular, indefinite backache, which she naturally attributed to uterine trouble. Later, her urine became offensive, and troubled her more. She lost flesh, and seemed to go steadily down-hill; had occasional fever, and her case was diagnosed by several doctors as malarial. At the time of my first visit, which I made at the request of her physician, these symptoms had continued with increasing severity; her loin had become swollen, tender, and deep fluctuation was present. Additionally, she suffered from constipation, lameness of limb on same side, which was contracted, and was unable to sit or stand erect. Urine was acid, contained mucus and pus, and was voided five or six times during night. There was no evidence of spinal disease or of any other viscera.

Peri-nephritic abscess was diagnosed and verified by aspiration. Free incision was made, releasing a pint or two of offensive but not urinous pus. No calculous hardness, enlargement, or disease of the kidney substance was evident from a digital exploration. Drainage and irrigation was instituted, and for a time she did well, when symptoms appeared pointing to pus retention. The opening was then enlarged, some adhesions and pus sacs broken away, and the kidney again thoroughly explored, without discovering any change in its condition—since which time, I am informed, she has been doing better, but her general condition is bad and the prognosis serious.

4. This case was a man—forty-six—admitted to the Sisters' hospital in an almost dying condition. High temperature, delirium, dyspnœa, expectoration of large quantity of pus. Under stimulants, he managed to live a few days, and stated that he had been sick for several weeks, but only had the cough very bad for a few days, and for which his friends urged him to be taken to the hospital. He was too sick to obtain much history from, and from the physical signs I diagnosed pneumonia, followed by pulmonary abscess. The day of his death, my attention was called to a fluctuating swelling in his groin, which, however, I considered would be best studied at the autopsy.

*Post-mortem* showed large abscess cavity back of left kidney, which was itself normal, the pus having burrowed upwards through the diaphragm into the lung, and downward in sheath of the psoas to Scarpa's triangle.

A diagnosis early in the disease is always difficult and often impossible. Sub-acute, slowly-developing cases, when the local symptoms are not pronounced, are apt to be confounded with enteric or malarial fever, from which, however, they are to be distinguished by the gradual development of abdominal symptoms, eruption, and peculiar temperature in the one, and influence of ante-periodics in the other.

When pain is the prominent symptom, lumbago may be simulated, but it is usually bi-lateral, the tenderness more marked by pinching the muscles; there is no fever or contraction of the psoas, and as the case progresses, the diagnosis can be made positive by noting the influence of treatment and the further development of characteristic one-sided features.

Neuralgia, pain, etc., not due to known pathological changes, are paroxysmal, and occur in neurotic individuals.

The pain of early spinal disease is relieved by suspension, and the disease defined by the hot sponge and pressure over individual vertebræ—later by curvature.

" Gibney "[5] reports in *American Journal of Medical Science*—78 and 79—a number of cases of peri-nephritic abscess in children, where the symptoms simulated coxalgia. In such cases, the diagnosis is to be made by observing the absence of pain at hip on motion, tenderness over trochanter, wasting of limb, etc., and, additionally, by the presence of lumbar, tenderness, difference in temperature of the two loins.

When the local condition is well marked, these abscesses are to be differentiated from numerous other swellings liable to occupy this region.

Hepatic tumors are influenced by respiration; splenic are more apparent anteriorly—both are non-febrile. Fecal accumulations are influenced by purgatives.

Peri-typhlitis has been confounded, and occasionally, when burrowing of pus has occurred, it is extremely difficult to decide. Both are local inflammatory diseases with general symptoms, and are apt to develop and pursue indistinct courses. In the one, however, the local manifestations are chiefly behind and above; in the other, in front and below; and the histories show their development from these different points. Flexion of thigh is absent in one, and œdema of limb liable to occur in the other, and, when opened, peri-typhlitic abscess may contain stercoracious pus and and even gas.

From psoitis it is distinguished by there being more trouble in front than behind, the pain and swelling being nearer the middle line than in one drawn from the axilla to crest of illium, as in lumbar abscess. Psoas abscess, depending on caries of vertebræ, is accompanied by spinal symptoms, and pus contains bone detritus.

Disease of the kidney itself may be mistaken for the abscess in question. It may precede, co-exist and be the cause. Most of them, however, cause an enlargement more appreciable through the abdominal walls than through the loins, are not marked by pain, fever and contraction of the limb, and the urine furnishes characteristic information.

Briefly, *cancer* is non-febrile and accompanied by cachexia hematuria and family history.

*Hydro-nephrosis* pertains to a non-inflammatory fluctuating tumor, with a definite history of gravel or calculus, colic, blood, urinary obstruction, no pus, and variation in quantity of urine excreted.

*Pyo-nephrosis* is febrile, but less painful, the swelling a gradual growing circumscribed tumor, and there is absence of contraction of psoas.

*Tubercular kidney*, if not present in a generally tuberculous subject, is usually accompanied by manifestations of the same disease lower down, and, in addition to pain swelling in lumbar region, pus and cheesy matter appear in the urine, in which the bacillus might be found.

The primary or secondary nature of these abscesses is determined by the clinical history which may precede each, and which has been outlined. The prognosis is good in primary cases, if early evacuation takes place externally, either spontaneously or by incision; but as there is little tendency to such evacuation, burrowing has often occurred before surgical interference, and in direct proportion to its extent and the coincident constitutional impairment, the prognosis becomes doubtful and even grave. In secondary cases, the forecast is upon the same lines, plus the gravity of the pre-existing disease. Often a cure is incomplete, a fistula remaining, which may be permanent, or heal only after some years.

Efficiency of treatment depends largely upon early evacuation.

Peri-renal inflammation can occasionally be checked if recognized before suppuration. Under such circumstances, antiphlogistic treatment is to be followed—local blood-letting, poultices, blisters, purgatives, anodynes, etc.

Suppuration demands free incision as soon as its existence is detected, and the aspirator used, if only suspected. Free early incision saves life, and hastens recovery. Even if there be only tenderness and tension, with great pain, an incision is indicated, relief following, and, later, pus. The opening can be oblique or vertical, and be made boldly or, what is better, by a gradual dissection—Hilton's method. The escape of matter should be followed by thorough exploration of kidney, disinfection, drainage, irrigation. One precaution is to be borne in mind: not to permit too early closure; particularly in secondary cases, the granulations should force the drainage-tube out. Remaining fistules are to be treated upon ordinary principles and, in any event, appropriate constitutional attention is given in way of food, stimulants, tonics, etc.

